# JujubeBruiseNet: A high-resolution image dataset for bruise detection in *Ziziphus mauritiana*

**DOI:** 10.1016/j.dib.2025.112031

**Published:** 2025-09-03

**Authors:** Md Arham Tabib, Sumyia Sabrin Liza, Md Mizanur Rahman

**Affiliations:** Department of Computer Science and Engineering, Faculty of Science & Information Technology, Daffodil International University, Birulia, Dhaka 1216, Bangladesh

**Keywords:** Image dataset, Bruise detection, Jujube dataset, Fruit dataset, Image classification, Agriculture, Computer vision, Deep learning

## Abstract

The article presents JujubeBruiseNet, a high-resolution image dataset designed for bruise detection in *Ziziphus mauritiana* (jujube) fruits. *Ziziphus mauritiana* is a seasonal fruit often found in late summer to early fall. The bruise detection in this fruit is crucial for post-harvesting, fruit processing, and food packaging. Manual detection of bruises is time-consuming and often leads to inaccuracy. Therefore, developing a novel classification model is essential, which will immediately recognize bruises in the fruits and, as a result, decrease human effort, expenses, and production time in the agriculture sector. The dataset contains a total of 1464 original photos categorized by two classes labelled Healthy and Bruised. We collected the fruit from the local market and fields near Savar, Dhaka, Bangladesh, with the help of domain experts in the period from 10th March to 20th March 2025. To reduce outside variations and provide uniformity, the photos were taken under precisely controlled lighting. This article offers a major dataset for researchers to develop effective quality assessment models for post-harvesting fruit sorting and classification. Convolutional neural networks (CNNs) and other computer vision models can be trained exclusively using this dataset to increase the precision of agricultural product bruise recognition. The dataset can facilitate research in computer vision-based agricultural monitoring and fruit quality evaluation, openly accessible on Mendeley Data, link: JujubeBruiseNet: A Dataset for Bruise Detection in Ziziphus mauritiana - Mendeley Data

Specifications Table

**Table:** 

Subject	Agriculture Science & Computer Science
Specific subject area	Image Classification, Image Recognition, Deep Learning, and Computer Vision.
Type of data	Image
Data Collection Technique	The data was collected from 10th March to 20th March 2025. We collected both healthy and bruised jujube from the local market and field. The Samsung Galaxy Note8 with 12 MP resolution, a large sensor with bright f/1.7 aperture, and a 12 MP 2x zoom camera with optical stabilization were used to capture the images.
Data Format	Jpg
Data source location	Location: Local shops and field Zone: Savar, Dhaka Country: Bangladesh
Description of data collection	The samples had never been previously treated in any previous study project. We gathered all of the fruit photos with the help of a domain expert from an agricultural organization.
Data accessibility	Repository name: Mendeley Data Data identification number: 10.17632/3mtdhrwgfr.5 Direct URL to data: JujubeBruiseNet: A Dataset for Bruise Detection in Ziziphus mauritiana - Mendeley Data

## 1 Value of the Data

• The JujubeBruiseNet dataset enables high-resolution images of *Ziziphus mauritiana*(jujube) fruit with two different classes healthy and bruised providing an opportunity for researchers on bruise detection in agriculture products.
• The data is formatted with clear-cut class-wise folders and has heavy metadata (labels) so that it can be natively incorporated in classification pipelines.
• The dataset delivers a broad and appropriate range of samples to efficiently train, validate, and test deep learning models, with 732 original images per class.
• Data augmentation techniques such as rotation, flipping, brightness/contrast, and zoom simulate real-world conditions and increase model robustness.
• This dataset can be used for enhancing post-harvest efficiency by utilizing various computer vision applications such as object detection, quality grading, and automated sorting in agriculture.
• Convolutional Neural Networks (CNNs) and other machine learning methods can be evaluated using this dataset, allowing for comparative analysis and reliable study.

## 2 Background

Fruit has nutritional value and it also plays a vital role in our economy. Bruise in fruit reduces the nutritional value. Fruit quality, market value, and customer happiness, are all greatly impacted by this prevalent post-harvest problem. Computer vision, as well as machine learning-based approaches, have emerged as promising solutions for automating fruit quality assessment to overcome the time-consuming, labor-intensive, and human prone to mistakes nature of the traditional inspection process [1]. *Ziziphus mauritiana* is the scientific name of a commonly cultivated species of jujube [2], known as Kul or Apple Kul in Bangladesh. JujubeBruiseNet's main goal is to support automated fruit quality analysis research [3]. The subjective, time-consuming, and uneven nature of traditional manual inspection techniques highlights the need for an effective, automated solution. To identifying the presence of bruises [4], this dataset has a great deal of potential for use in quality evaluation, illness diagnosis, and defect categorization, all of which advance the field of precision agriculture. There are many existing datasets for quality evaluation [5], classification [6], maturity grading [7] or disease detection [8] of different fruits. Especially fruits like Jujube which are very vulnerable in the time of production from the field to consumers’ hands, any kind of damage like bruises are very common. JujubeBruiseNet was created to solve this problem by offering a trustworthy dataset for deep learning and machine learning models that are intended to detect bruises, especially for these fruits. The collection includes high-resolution photos taken in carefully regulated lighting, guaranteeing uniformity and emphasizing surface-level characteristics essential for classification tasks. Data augmentation improves model generalization by increasing variability and simulating real-world settings. The dataset is a useful resource for agricultural automation and computer vision research on its own [9]. By providing a standard dataset for bruise identification, it also supports related research by facilitating comparisons between various methods. We hope that making this dataset freely accessible will help the agricultural technology community advance fruit quality assessments and encourage repeatable research.

## 3 Data Description

**Fig. 1:**
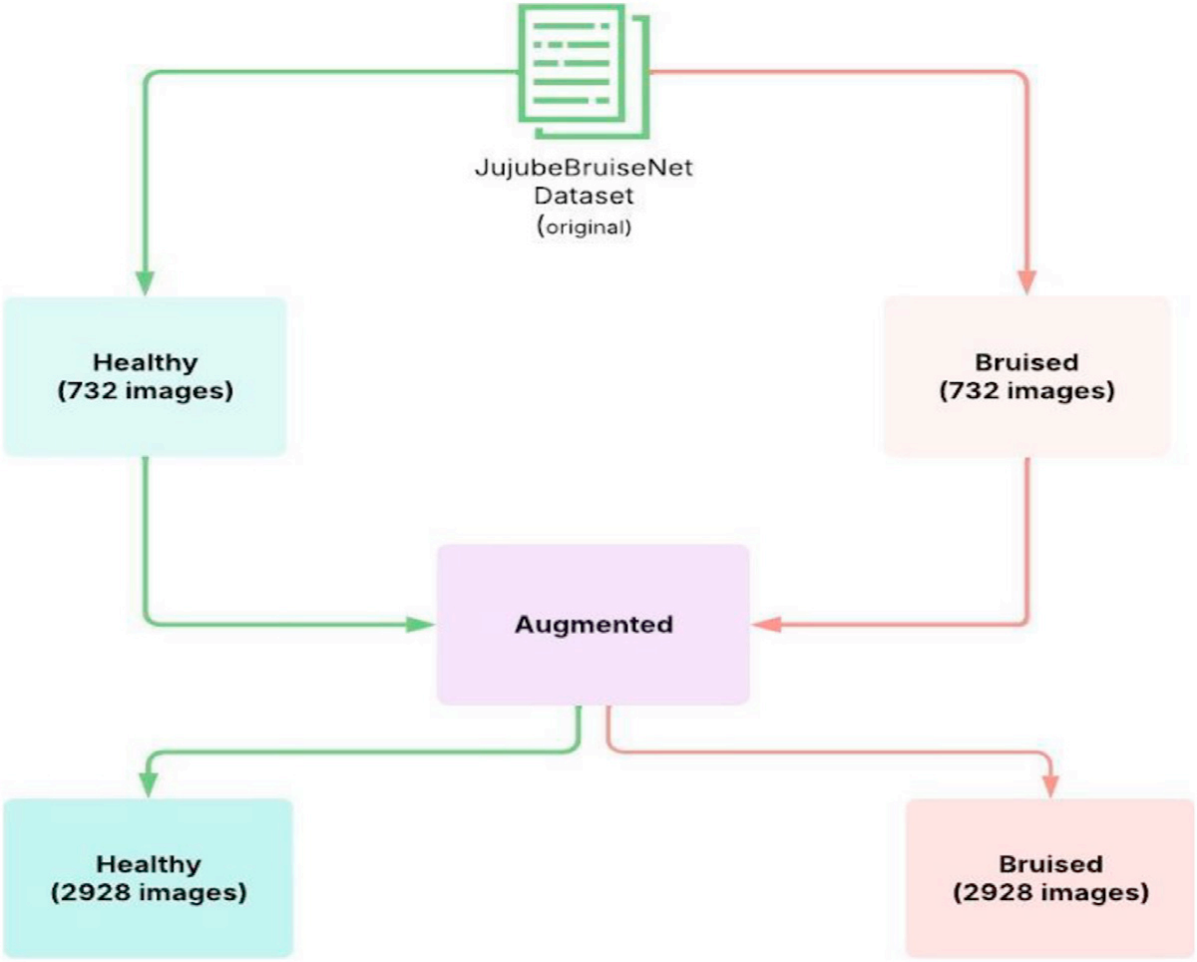
Structured folder layout of the JujubeBruiseNet dataset.

The JujubeBruiseNet dataset is organized into two primary folders shown in Fig 1: Healthy and Bruised representing two distinct classes of *Ziziphus mauritiana* (jujube) fruits. Each folder contains original high-resolution images and a corresponding set of augmented images. The folder contains images labelled like healthy/healthy_1.jpg, bruised/bruise_1.jpg, augmented/healthy/aug_0_healthy_1.jpg, augmented/bruised/aug_0_bruise_1.jpg. The dataset is structured to facilitate easy integration with machine learning and deep learning frameworks. The number of images in each class are same so there is no chance for model bias.

Table 1 is a concise overview of the JujubeBruiseNet dataset, describing its organization and key technical characteristics. It summarizes the number of images in the original and generated datasets for the two categories Healthy and Bruised and describes the file type, resolution, and color mode. Organized presentation allows readers to quickly assess the size, organization, and suitability for computer vision tasks such as classification and bruise detection of the dataset.

**Table 1:** Data summary table.

Attribute	Description
Total Raw Image	1464 (732 healthy and 732 bruise)
Total Augmented Images	5856 (2928 healthy and 2928 bruise)
Combine Dataset	7320 images
Image File Format	JPGE(.jpg)
Image Resolution	512 × 512
Color Mode	RGB
Augmentation Technique	Rotation, Brightness, Contrast, Flip

This table provides us a clear view between the healthy and bruised jujubes.

**Fig. 2:**
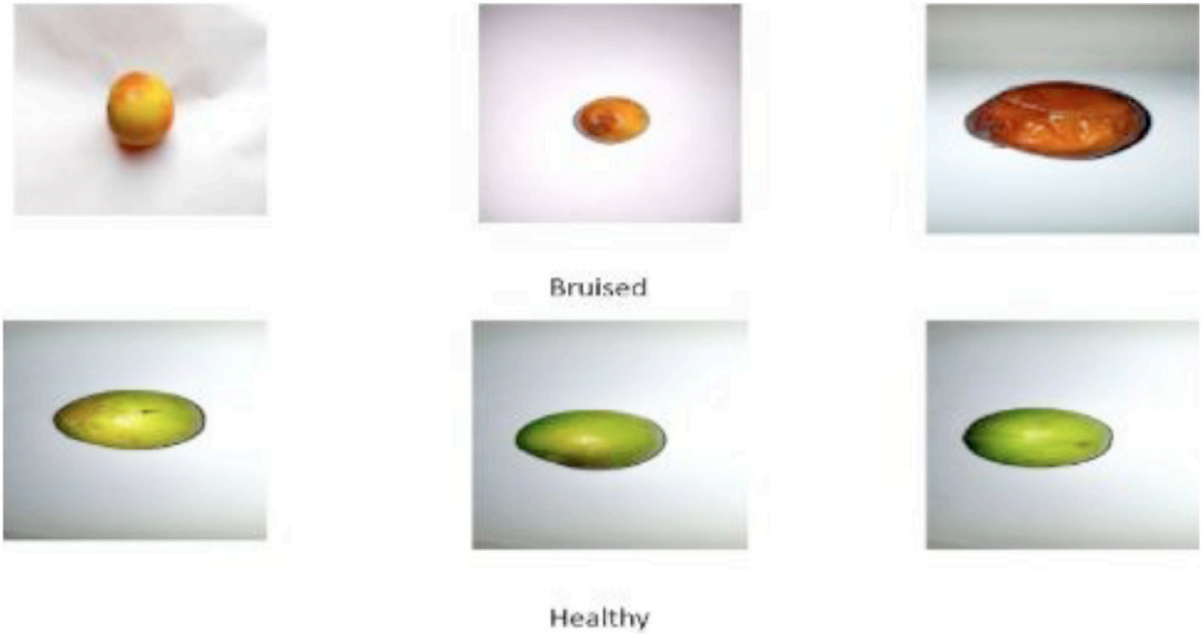
Sample dataset.

The Fig. 2 shows us the sample data of both classes collected for the dataset. It contains 3 images of bruised jujube and 3 images of healthy jujubes.

### 3.1 Contents and structure

• **Jujube collection:** Local markets and fields were visited to collect jujube. The raw dataset consists of 732 images in each class. After collecting the jujubes, they are categorized into bruise and healthy class with the help of a domain expert. Table 2 shows the raw data of two different classes.

**Table 2:** Class description.

Class	Description	Image
Healthy	Jujubes are green when they are unripe. They become yellow-green with reddish–brown as they start to ripe. The fruit is all red when it is fully ripe. From yellow-green to completely red, we can eat them at any stage of development; the redder they are, the sweeter they will taste. They are crisp and tasty, with a texture similar to an apple.	
Bruised	The surface of bruised jujube fruit frequently exhibits obvious damage or discolor. Initially appearing as, a dark reddish-to-black mark, the bruised region may eventually deepen into a dark brown or reddish-brown hue. The fruit's peel may thicken around the injured area, and these damaged spots may become sunken. Internal injury is typically indicated by the tissues beneath the bruised surface turning dark brown. In comparison to the rest of the apple, the bruised area may feel softer overall. The size of the bruise may determine whether the jujube's unaffected portions are still edible despite the blemish.
• **Image collection:** The Samsung Galaxy Note 8 device was used to capture the photo. The device has a 12 MP resolution with a large sensor with bright f/1.7 aperture and a 12 MP 2x zoom camera with optical stabilization allowing us to capture high-resolution images. Jujubes were stored in the center of a white paper. The room temperature was controlled. The flash of the camera was not utilized in capturing the images. Jujubes were exposed to a 20 W room LED light at a mean distance, no other light setting was made. After clicking the photo, we used a cam Scanner app to scan the images making it clear and noise-free.
• **Augmentation:** Each image was assigned to an augmentation process where several operations were performed to ensure data consistency and diversity. Table 3 provides a clear overview about the augmentation process we have used on this dataset. We used:
  ○ **Horizontal Flip:** Flips the image horizontally. Creating a mirror version and increases the viewpoint variation. As fruits do not have inherent directional bias so horizontal flip was used.
  ○ **Random Brightness and Contrast Adjustment:** Adjusts the image's brightness and contrast by applying random values within specified ranges. Alpha was used for image contrast, randomly scales pixel intensities (0.7 to 1.3 range) and beta was used for brightness, randomly shifts pixel values (−50 to +50 range)
  ○ **Random Rotation:** It introduces viewpoint variation through in place rotations. Rotates the image by a random angle between −30 and 30 degrees. It may help models to recognize objects from different angles.
  ○ **Gaussian Blur:** A Gaussian blur simulates varying focus on the conditions and reduced the high frequency noise. We applied 5 × 5 Gaussian kernel for smoothing. Standard deviation was automatically calculated from kernel size.

**Table 3:** Result after augmentation.

The table shows jujube images are converted using the augmentation techniques widely used to improve model performance in deep learning. Each row presents a class. The first rows present the healthy class after augmentation and the second row presents the bruise class after augmentation.

The augmentation process was applied using python code in Jupiter notebook. The python library function PIL (Python Imaging Library) used for loading the image and converting RGB format to ensure the consistency of color channels. Torchvision Transforms is used for other augmentation steps.

The following algorithm was followed to apply the augmentation:

Step 1 Load the original image from the dataset.

Step 2 Save the original image in the “Original” folder for baseline reference.

Step 3 Apply random brightness adjustment to the original image.

Step 4 Apply random contrast adjustment to the image from Step 3.

Step 5 Apply Gaussian blur to the image from Step 4.

Step 6 Save the resulting image as the first augmented version.

Step 7 Apply a random rotation (e.g., within ±30 degrees) to the original image.

Step 8 Save the rotated image as the second augmented version.

Step 9 Apply a horizontal flip to the original image.

Step 10 Save the flipped image as the third augmented version.

Step 11 Repeat Steps 1–10 for all images in the dataset.

The Table 4 presents the data statistics before and after augmentation. We collected 327 healthy fresh jujubes and 283 bruised jujubes and captured 732 images (multiple image per fruit) each class from the collected jujubes to balance the classes. In augmentation process we generated 3 images from one image which made the augmented image count 2928 per class.

**Table 4:** Data stats.

Class	Jujube Collected	Original Image	Augmented Image	Total image
Healthy	327	732	2928	3660
Bruised	283	732	2928	3660
Total	610	1464	5856	7320

Table 5 compares our proposed dataset with some existing fruit image datasets. While earlier studies by Sultana et al. [10], Abedin et al. [11], and Saha et al. [12] are focused on classes of fresh and rotten fruits, none of them included bruise samples.

**Table 5:** Comparison with available dataset on fruits.

Dataset	Class	No of Fruit	Number of Original Image	Number of Augmentation Image	Fresh	Bruise
Sultana et al. [10]	16	8	3200	12,335	✓	×
Abedin et al. [11]	10	10	3173	×	✓	×
Saha et al. [12]	7	7	5470	28,500	✓	×
This Dataset	2	1	1464	5856	✓	✓

## 4 Experimental Design, Materials and Methods

**Fig. 3:**
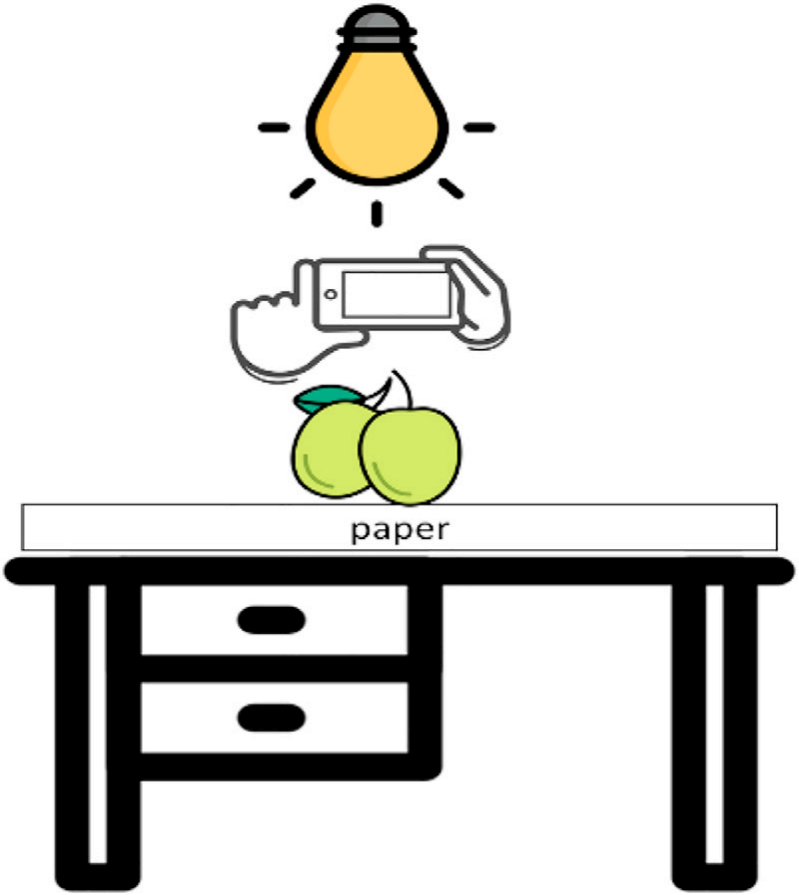
Image capturing Setup.

**Fig. 4:**
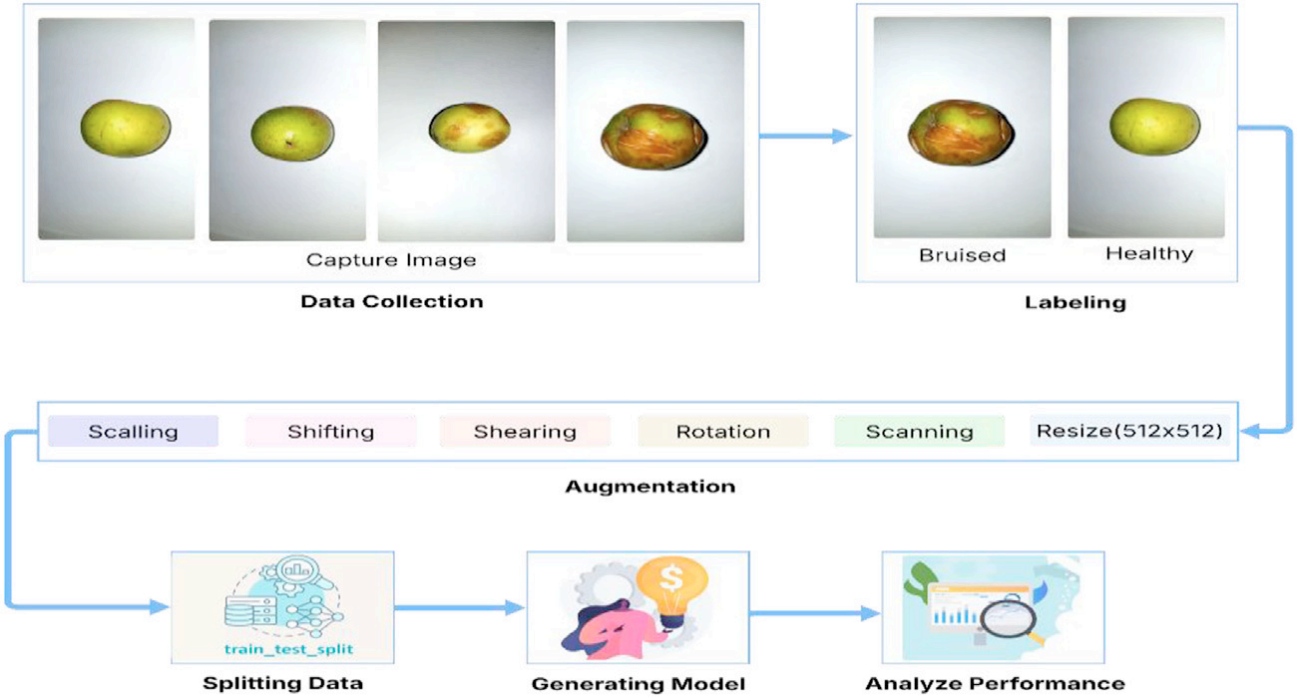
Methodology.

The information is intended for a thorough study to identify bruises in jujube, enabling researchers to help control crop quality after harvest. To maintain even lighting and improve the visibility of surface textures, a controlled environment was made available with the assistance of a single 20-watt LED room light to minimize reflections and shadows. The photographs were captured in high resolution by a Samsung Galaxy Note 8 phone, which features a 12 MP camera. Subcontinental nations including Bangladesh, Malaysia, and India are home to the fruit. Therefore, the dataset fills in the gaps in the data source allowing researchers to develop wonderful deep learning models. Fig. 4 provides a proper methodology to utilize this dataset for research purposes. At first, we gathered fresh *Ziziphus mauritiana* fruits from farms and local shops. Total 610 jujube fruit was collected and then the selection of healthy fruits was based on their stable texture and not having obvious damage. In order to replicate actual handling damage, bruised apples were collected naturally occurring in the texture. A proper camera was used to capture the images and then label them. Augmentation techniques were applied to increase dataset diversity and improve the generalization ability of classification models. Table 4 illustrates the data after applying the augmentation process. The data is ready to proceed with applying the model. One can apply various models to the dataset, analyze the model performance, and deploy useful applications to detect bruises in jujube fruit. This experimental setup ensures that JujubeBruiseNet is a comprehensive and diverse dataset, supporting the development of robust bruise detection models in agricultural automation. Table 6 provides a detailed summary of experimental design. Images were captured using one 20 W LED light placed 90° above of the fruits at a distance of 80 cm with a white background using an A4 paper.The Fig. 3 provides a detailed knowledge about the image capturing setup.

**Table 6:** Experimental setup summary for JujubeBruiseNet dataset.

Parameter	Description
Camera Specification	Samsung Galaxy Note 8, 12 MP rear camera
Lighting Setup	One LED room light, 20 W, placed overhead
Environment	Indoor setup with white paper backgroud
Data Collection Sites	Local firms and markets in Savar, Bangladesh
Bruise Criteria	Minimum 5 mm visible discoloration
Augmentation Tool	Torchvision
Augmentation Strategy	Sequentially per image

**Fig. 5:**
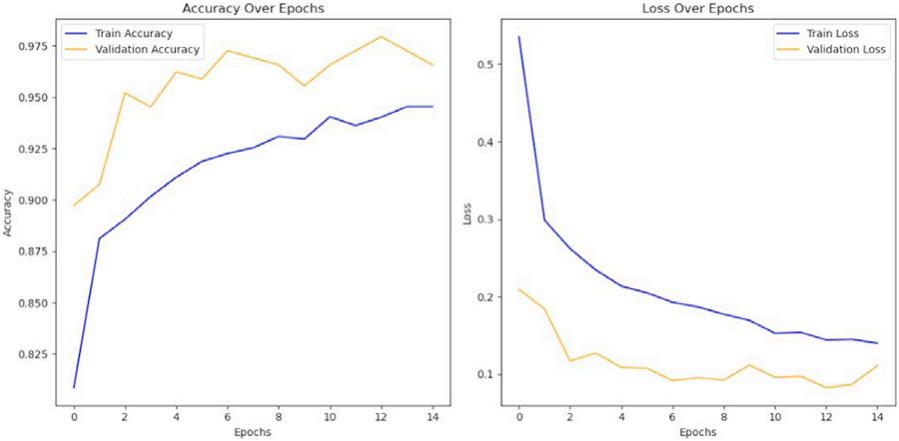
Accuracy Loss Graph of VGG16 applied on jujubeBruiseNet dataset.

Advanced deep learning models like VGG-16, Fast R-CNN, Mask R-CNN, DenseNet-121, and RestNet were efficiently trained and trimmed. The VGG-16 performed well in the dataset having almost a 99 % accuracy. Fig. 5 shows the training and validation accuracy of this dataset on VGG-16 and Table 6 contains the overall model performance. The data was split into 80 % train and 20 % validation subsets using function with a fixed random seed (123) for reproducibility. A regular camera was used to capture the images and then label them. Augmentation was done using Torchvision-based preprocessing pipeline and applying transformation like rotation, zoom, flip, and brightness change. Random augmentation was applied to every image with a random combination of transformations in every run in sequence.

The accuracy loss graph shown in Fig. 5 describes how well the model performs on the dataset. The effectiveness of a deep learning model's training and validation across nine epochs using the JujubeBruiseNet dataset. The Training and Validation Accuracy is plotted on the right, and the Training and Validation Loss is plotted on the left. Starting at about 1.4, the training loss rapidly decreases over the first several epochs until settling at values close to zero after epoch 7. Throughout the training phase, the validation loss stays constant at a low level, suggesting no overfitting and strong generalization. By epoch 6, the training accuracy has increased from about 82 % at epoch 1 to over 92 %. Strong generalization on unseen data is indicated by the validation accuracy, which maintains consistently high performance, peaking close to 97 % after beginning at 90 %. These charts demonstrate the dataset's durability and the implemented model's effectiveness. The JujubeBruiseNet dataset appears to be a good fit for binary classification tasks involving bruise identification, as evidenced by its quick convergence and small difference between training and validation measures (Table 7).

**Table 7:** Models overall performance on the dataset.

VGG16				
	Precision	Recall	F1-score	support
Bruised	0.94	1.00	0.97	148
Healthy	1.00	0.93	0.96	144
				
Accuracy			0.97	292
Macro Avg	0.97	0.97	0.97	292
Weighted Avg	0.97	0.97	0.97	292

The content displays performance measures of a deep learning model, specifically a model named VGG16. There are two different tables, each showing the result of the classification task of the model. These tables demonstrate how well the model performs when classifying Bruised and Healthy fruit, with extremely high accuracy and consistency across metrics.

## Limitations

The JujubeBruiseNet dataset has limitations even if it provides a large collection of photos for *Ziziphus mauritiana* fruit bruise detection. First off, while the dataset was gathered in controlled lighting settings, it might not accurately reflect real-world situations where environmental elements and lighting shift greatly. All images were captured solely from Savar, Dhaka under controlled lighting, limiting the generalizability of the dataset across other regions and natural field environments. Future work can include expanding the dataset with geographically diverse samples, multiple region based and various lighting setup samples, other types of jujubesand varied environmental setups to achieve better generalizability. **We also plan to extend the dataset in future iterations by incorporating images captured under varied lighting conditions, across different geographic regions, and from multiple jujube cultivars to promote broader generalizability and community-driven enhancements.** As a result, when used in natural environments, models trained on this dataset may need further fine-tuning or transfer learning. Second, because the dataset only looks at surface-level bruises, it can miss interior damage that is hidden from view. Future datasets can also explore emerging imaging technologies, such as hyperspectral imaging or X-ray, to detect subsurface or internal bruising not visible to the naked eye, boosting defect detection ability. This might reduce the models' ability to identify latent flaws. Furthermore, even while the augmentation approaches increase the diversity of data, they may unintentionally add unrealistic variations that do not adequately reflect conditions in the real world. Finally, the dataset contains images of a single fruit species, limiting its generalizability to other fruit types or agricultural products. Future datasets could benefit from multi-species inclusion and more diverse environmental conditions to enhance model robustness and real-world applicability.

## Ethics Statement

The dataset used in this work was collected ethically, ensuring that no animals, plants, or the environment suffered any harm. The photographs were properly taken, and the required permissions were obtained where needed. The dataset is only intended for academic and research purposes, with the aim of improving agricultural disease detection and management.

## Credit Author Statement

**Md Arham Tabib:** Conceptualization, Data curation, Methodology, Visualization, Project administration, Writing - original draft. **Sumyia Sabrin Liza:** Data Curation, Visualization, Labelling, Writing - original draft. **Md Mizanur Rahman:** Supervision, Formal analysis, Methodology, Writing - review & editing.

## Data Availability

Mendeley DataJujubeBruiseNet: A Dataset for Bruise Detection in Ziziphus mauritiana (Original data).
